# An automated hybrid approach via deep learning and radiomics focused on the midbrain and substantia nigra to detect early-stage Parkinson’s disease

**DOI:** 10.3389/fnagi.2024.1397896

**Published:** 2024-05-20

**Authors:** Hongyi Chen, Xueling Liu, Xiao Luo, Junyan Fu, Kun Zhou, Na Wang, Yuxin Li, Daoying Geng

**Affiliations:** ^1^Academy for Engineering and Technology, Fudan University, Shanghai, China; ^2^Department of Radiology, Huashan Hospital, Fudan University, Shanghai, China; ^3^Institute of Functional and Molecular Medical Imaging, Fudan University, Shanghai, China; ^4^China Center for Shanghai Intelligent Imaging for Critical Brain Diseases Engineering and Technology Research, Shanghai, China

**Keywords:** Parkinson’s disease, radiomics, machine learning, convolutional neural network, classification

## Abstract

**Objectives:**

The altered neuromelanin in substantia nigra pars compacta (SNpc) is a valuable biomarker in the detection of early-stage Parkinson’s disease (EPD). Diagnosis via visual inspection or single radiomics based method is challenging. Thus, we proposed a novel hybrid model that integrates radiomics and deep learning methodologies to automatically detect EPD based on neuromelanin-sensitive MRI, namely short-echo-time Magnitude (setMag) reconstructed from quantitative susceptibility mapping (QSM).

**Methods:**

In our study, we collected QSM images including 73 EPD patients and 65 healthy controls, which were stratified into training-validation and independent test sets with an 8:2 ratio. Twenty-four participants from another center were included as the external validation set. Our framework began with the detection of the brainstem utilizing YOLO-v5. Subsequently, a modified LeNet was applied to obtain deep learning features. Meanwhile, 1781 radiomics features were extracted, and 10 features were retained after filtering. Finally, the classified models based on radiomics features, deep learning features, and the hybrid of both were established through machine learning algorithms, respectively. The performance was mainly evaluated using accuracy, net reclassification improvement (NRI), and integrated discrimination improvement (IDI). The saliency map was used to visualize the model.

**Results:**

The hybrid feature-based support vector machine (SVM) model showed the best performance, achieving ACC of 96.3 and 95.8% in the independent test set and external validation set, respectively. The model established by hybrid features outperformed the one radiomics feature-based (NRI: 0.245, IDI: 0.112). Furthermore, the saliency map showed that the bilateral “swallow tail” sign region was significant for classification.

**Conclusion:**

The integration of deep learning and radiomic features presents a potent strategy for the computer-aided diagnosis of EPD. This study not only validates the accuracy of our proposed model but also underscores its interpretability, evidenced by differential significance across various anatomical sites.

## 1 Introduction

Parkinson’s disease (PD) is a neurodegenerative disease characterized by bradykinesia, rest tremor, and rigidity. The pathological changes of PD are characterized by a progressive loss of neuromelanin containing dopaminergic neurons and an increase in iron deposition in substantia nigra pars compacta (SNpc) (Damier et al., 1999; Ward et al., 2014). At present, neuromelanin-sensitive magnetic resonance imaging (NM-MRI) can be used to identify the reduction of neuromelanin (Taniguchi et al., 2018; Gaurav et al., 2021) and the quantitative susceptibility mapping (QSM) could reflect the iron deposition of PD patients in SNpc (Guan et al., 2017). Furthermore, the short-echo-time magnitude (setMag) images, reconstructed using the shortest three TEs image from quantitative susceptibility mapping (QSM), are capable of indicating the decrease of neuromelanin in SNpc. This technique offers considerable promise for the diagnosis of PD (Liu et al., 2020).

However, detecting PD at an early-stage is challenging in clinical practice based on magnetic resonance imaging (MRI) images, since alterations in brain structure are particularly subtle. Takahashi et al. (2018) evaluated NM-MRI and QSM modalities to distinguish PD with AUC values of 0.86 and 0.68, respectively. Ogisu et al. (2013) achieved an AUC of 0.90 for differentiating early-stage PD from healthy controls(HCs) based on signal threshold measurements of SN volume, but sensitivity and specificity could not be well balanced. Pyatigorskaya et al. (2017) discriminated idiopathic rapid-eye-movement sleep behavior disorder (iRBD) based on NM-MRI images using SNpc volume and signal intensity with an accuracy of 0.86 and 0.79, respectively. Compared to that, the doctor’s visual accuracy was 0.81. Up to now, neither visual recognition nor semi-automatic analysis is fully satisfied. Therefore, we need to develop a more accurate and efficient diagnostic approach to identify and monitor patients with early-stage PD.

Recent studies (Salvatore et al., 2021) have demonstrated that radiomics analysis is an efficient method to help the diagnosis of neurodegenerative disorders like Parkinson’s disease. This method can offer large amounts of quantitative features indicating tissue heterogeneity and other information related to pathologic changes. Radiomics features are filtered to build machine learning models. In the study by Ren et al. (2021), radiomics features extracted from 95 PDs and 95 HCs were filtered by least absolute shrinkage and selection operator (LASSO) and achieved a predictive effect of AUC = 0.81 based on susceptibility-weighted imaging(SWI). Cao et al. (2020) used the least absolute shrinkage and LASSO to select radiomics features of rs-fMRI from 50 HCs and 70 PDs, and the trained support vector machine (SVM) achieved 85% accuracy. However, radiomics features are mainly extracted based on expert knowledge or certain rules, which makes the method challenging to 人incorporate new features or unusual data. Therefore, the radiomics approach lacks generalization and robustness.

Besides, deep learning (DL) is becoming increasingly important in medical image analysis. Compared with other methods, DL models can learn more complex and deeper features from raw data without additional processing (Anwar et al., 2018). Convolutional neural networks (CNNs), which come in a variety of models, are one of the most effective DL tools. Sivaranjini and Sujatha (2020) obtained an AlexNet whole brain model with 88.9% accuracy based on T2-weighted MRI using a transfer learning strategy. However, as a black box, CNN models can hardly comprehend the diagnosis they make. To solve this issue Le Berre et al. (2019) attempted to segment the SNpc region automatically and calculated its reduced volume directly. In the external validation set, the Dice coefficient for the SNpc segmentation reached 0.79 and the AUC for the diagnosis of PD reached 0.944. In the previous work, the CNN model was mainly trained with images of the entire head. Since the pathological changes of PD mainly occur in the brainstem, focusing the CNN model on the brainstem was thought to help diagnose (Bhan et al., 2021). Shin et al. (2021) used the YOLO v3 model to automatically detect and diagnose brainstem SNpc on susceptibility map-weighted MRI with an AUC of 0.94.

In this work, we endeavored to develop an automated hybrid model based on setMag images to detect early-stage PD, with radiomics and deep learning methodologies. First, radiomics features were extracted from the SNpc. Then, we obtained the brainstem region via the YOLO v5 detection model. Deep learning features of the brainstem region were extracted by a modified LeNet. Finally, we combined radiomics and deep learning features to develop a hybrid classification model for aided diagnosis of early-stage PD.

## 2 Methods and materials

### 2.1 Patients

A total of 65 healthy controls (HC) between 43 and 69 years old and 73 PD patients between 37 and 79 years old were recruited in this study. The HC sample had no history of neuropsychiatric or neurological diseases. Diagnosis of Parkinson’s disease was performed by movement disorder specialists in accordance with Movement Disorder Society (MDS) clinical diagnostic criteria (Postuma et al., 2015) for PD by a senior neurology specialist. The detailed inclusion criteria are provided in Supplementary material S1. It’s worth noting that the include PD patients were at early-stage with a oehn and Yahr stage (H-Y) ≤ 2 (Goetz et al., 2004). This study was approved by the Ethics Committee of Huashan Hospital (Center 1), Fudan University (approval No. KY 2016–214). The participants with QSM images in the Weifang Traditional Chinese Hospital (Center 2) were collected as the external validation. All patients or their guardians gave their informed consent to the utilization of their anonymized MRI images and clinical data for research purposes. MRI scanning parameters and detailed descriptions of the datasets are presented in Supplementary material S2.

### 2.2 Image post-processing and ROI delineation

SetMag images were reconstructed from QSM data. Within its multi-echo sequence, the first shortest echo time (TE) magnitude image has minimal T2*-weighted contrast and maximal T1-weighted contrast. As the echo time increases, the contribution of T2*-weighted contrast gradually increases (Liu et al., 2015). Therefore, we combined the magnitude images with the shortest three TEs and enhanced T1-weighted contrast, then acquired the setMag images, which was sensitive to neuromelanin. The detailed procession was followed our previous study (Liu et al., 2020).

**Figure 1:**
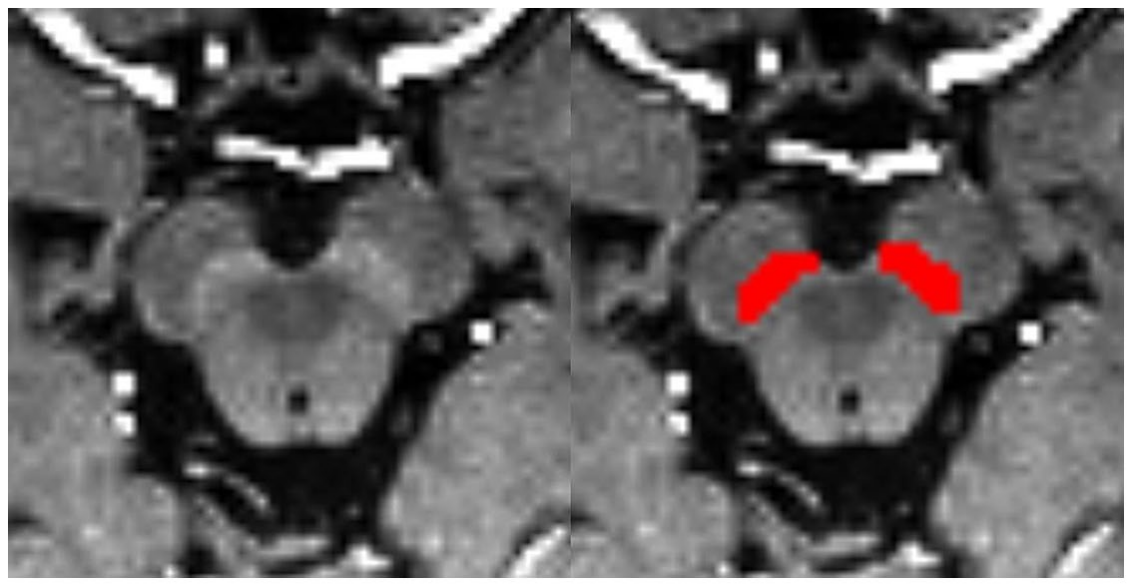
Bilateral segmentation of hyperintensity regions of interest (ROIs) on setMag images in SNc.

Bilateral hyperintensity regions of interest (ROIs) in SNpc were manually depicted on four contiguous axial slices of setMag images using ITK-SNAP software^1^ (Yushkevich et al., 2006) by a neuroradiologist1 (XL) with 10 years of experience. Meanwhile, 30 of all subjects were blind selected for secondary delineation by another neuroradiologist2 (NW) with 8 years of experience. The spatial overlap of each pair of ROIs segmented by two different raters (XL and NW) was compared using the Dice similarity coefficient (DSC) (Zou et al., 2004). Finally, a senior radiologist (YL) with 25 years of experience thoroughly reviewed and refined the delineated regions from neuroradiologist1, which were then used as the final ROIs for the following analysis. The most superior layer was in the axial section of the mammillary body, while the inferior section was positioned just above the pons where the inter-peduncular fossa opens up to the inter-peduncular cistern (Eapen et al., 2011; Figure 1).

In this study, the included 138 cases were divided into training-validation and test sets roughly by the ratio of 8:2. Specifically, the training-validation set contained 111 cases, while the test set included 27 cases. Since each case contained 4 images with ROIs, the training-validation set and the test set contain 444 and 108 images, respectively. We collected 12 healthy controls and 12 EPD from Weifang Traditional Chinese Hospital as an external validation dataset. The external validation set was used to evaluate the robustness of the established models.

### 2.3 Classification with radiomics feature

**Figure 2:**
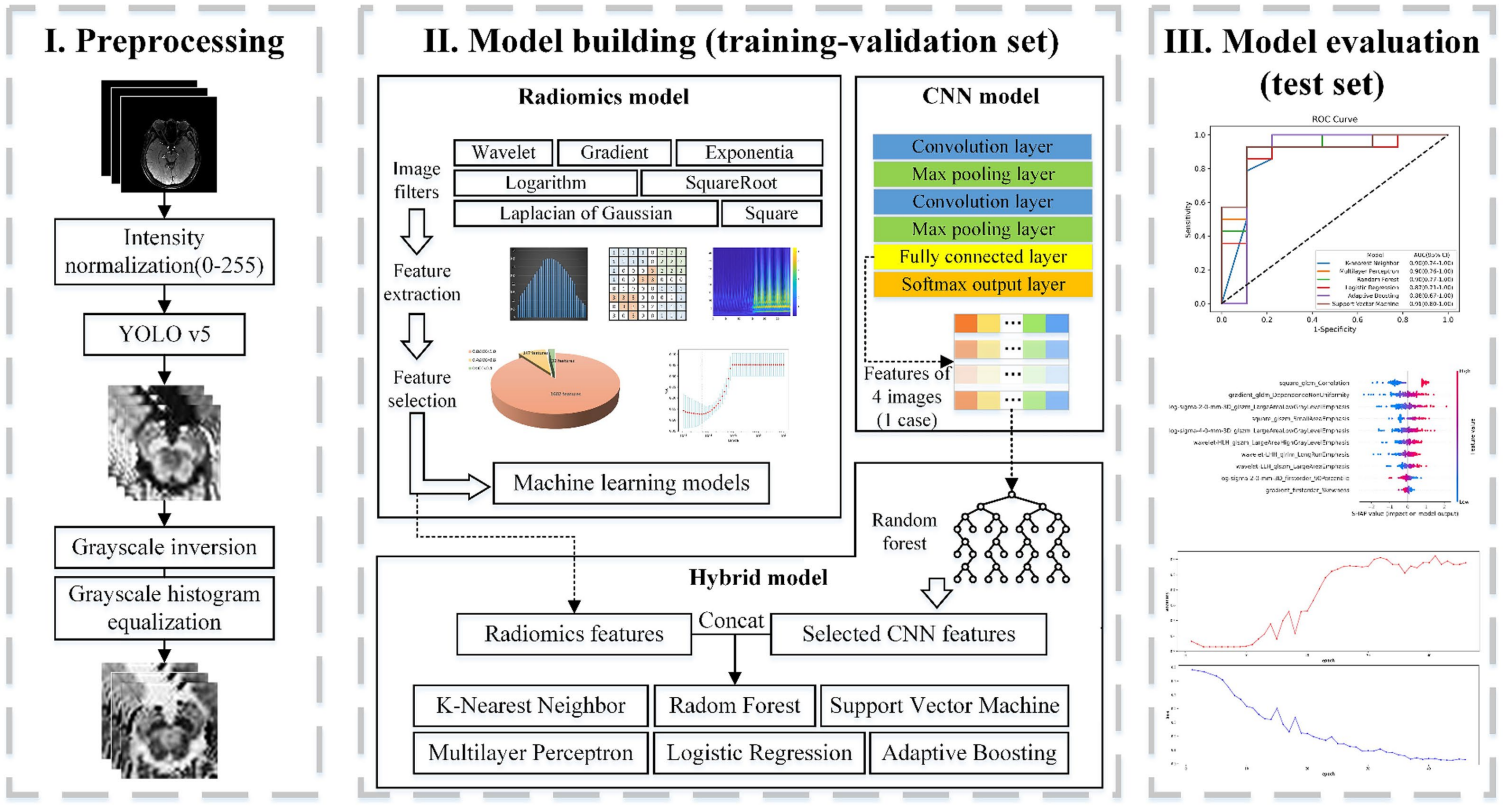
Flowchart of classification based on radiomics. Part I includes the preprocessing of the input setMag images with operations including intensity normalization, brainstem detection via YOLOv5, grayscale inversion, and grayscale histogram equalization. Part II indicates the establishment process of the radiomics feature-based model, the image-based CNN model, and the hybrid feature-based model. Part III displays model evaluation.

Following feature extraction from MRI images and subsequent selection, six machine learning models were trained: k-nearest neighbor (KNN), Random forests (RF), Logistic regression (LR), Support vector machine (SVM), Multilayer perceptron (MLP), and Adaptive Boosting (AdaBoost). The part of the radiomics model in Figure 2 depicts the processing flowchart. The model was first trained on the training-validation set with five-fold cross-validation and subsequently, the performance of the model was tested on the test set and the external validation set.

#### 2.3.1 Image preprocessing and feature extraction

To ensure consistency across MRI images’ ROIs for all patients, images were resampled and aligned to the same spacing, resolution, and alignment using nearest-neighbor resampling. Specifically, images were resampled to 0.5 × 0.5 × 1 mm^3^ voxels using the Analysis of Functional NeuroImages (AFNI) package.^2^ Radiomics features, compliant with the International Biomarker Standardization Initiative (IBSI), were then extracted using Pyradiomics.^3^ These features include six basic categories: (1) First-order statistics features; (2) Gray level co-occurrence matrix (GLCM); (3) Gray level dependency matrix (GLDM); (4) Gray level run length matrix (GLRLM); (5) Gray level size zone matrix (GLSZM); and (6) Neighboring gray-tone difference matrix (NGTDM). Additionally, each original image underwent seven transformations: wavelet, LoG, Square, SquareRoot, Logarithm, Exponential, and Gradient. All extracted radiomics features were normalized using the StandardScaler function in Scikit-Learn (Python).

#### 2.3.2 Feature selection, radiomics signature building, and validation

To address challenges arising from an excessive number of features, we employed the intraclass correlation coefficient (ICC) to filter out features with poor reproducibility. Specifically, features with an ICC greater than 0.8 were selected for subsequent analysis (Loeve et al., 2012). Moreover, we used the least absolute shrinkage and selection operator (LASSO) to keep the most crucial features.

Then, using six algorithms, we established prediction models based on the remaining features. Evaluation of model performance was conducted using various metrics, accuracy (ACC), sensitivity (SEN), specificity (SPE), positive predictive value (PPV), negative predictive value (NPV), F1-score, and the area under the curve (AUC) of Receiver-Operating-Characteristic (ROC) curve. Notably, the evaluation metrics were applied on a patient-wise basis, with the principle that a patient is diagnosed with EPD if three out of four images are classified as PD. Net reclassification improvement (NRI) and integrated discrimination improvement (IDI) were calculated to assess the enhancement of hybrid features for diagnosis. The relevant formulas can be found in Supplementary material S3. Finally, the Shapley Additive Explanation (SHAP) (Lundberg and Lee, 2017) approach was used to analyze the best model, providing insights into the role played by each clinical and radiomics feature in outcome prediction.

### 2.4 Classification with convolutional neural network

**Figure 3:**
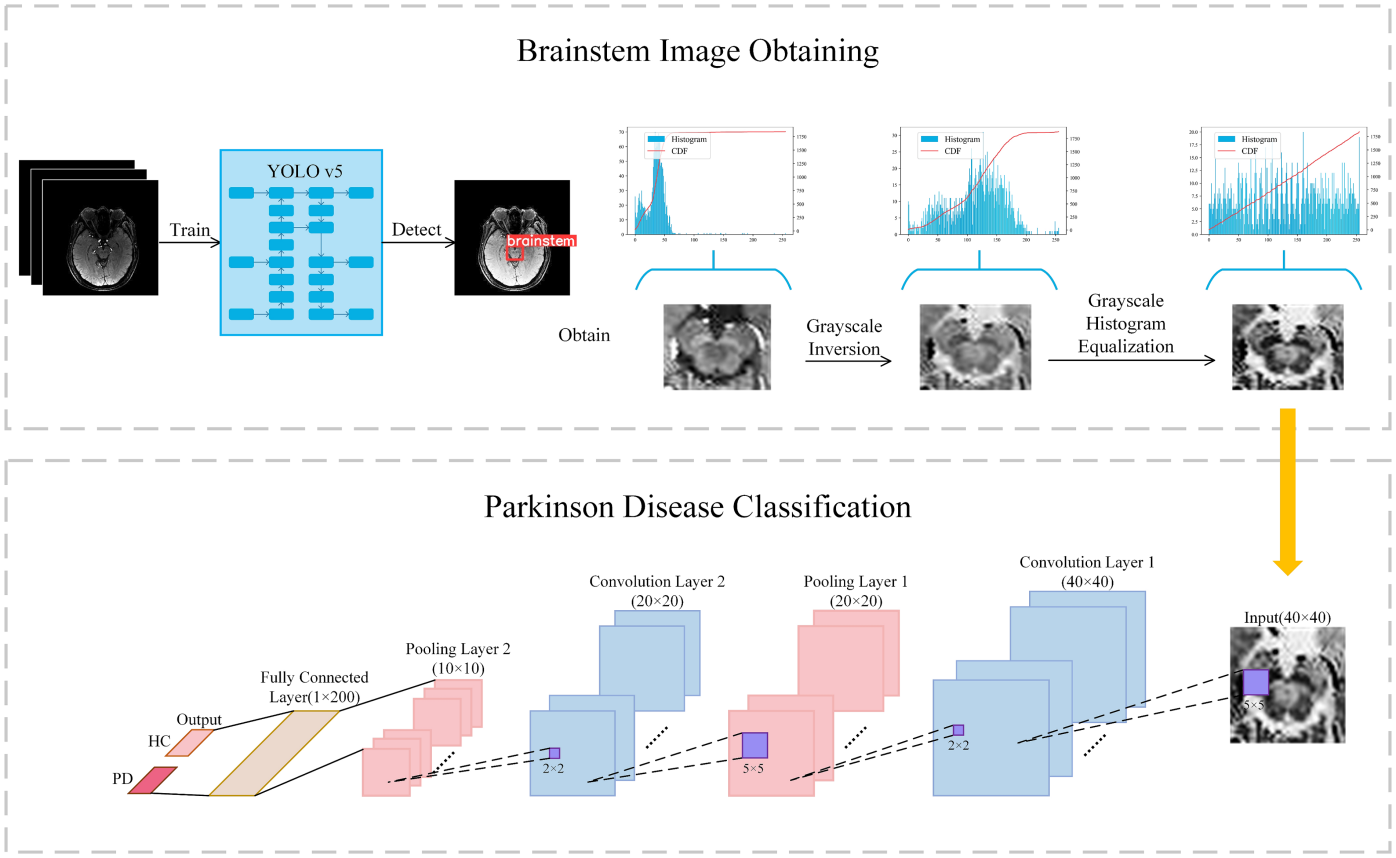
Flowchart of classification with convolutional neural network. Firstly, the brainstem image is obtained via YOLO v5 and preprocessed to enhance contrast. Secondly, the brainstem image was input in Lenet for classification.

In contrast to the radiomics-based approach, we proposed a convolutional neural network (CNN) to distinguish PD patients from healthy controls. It can be assumed that, compared to radiomics fixed feature extraction approaches, features generated by CNN provide superior issue solutions from a data-driven perspective because they are continuously optimized based on the classification accuracy of the training-validation set. At the same time, CNNs eliminate the arduous process of human annotation and have the ability to automatically extract brainstem areas and features. Our CNN-based flowchart is shown in Figure 3. The development of the CNN classification model consisted of two parts: (1) data augmentation and brainstem detection, and (2) construction and training of CNN.

#### 2.4.1 Data augmentation and brainstem detection

In light of previous research indicating the efficacy of data augmentation in enhancing model robustness, we applied data augmentation to all training data. Patch images were randomly scaled and rotated randomly within a small range, facilitating a tripling of the training image dataset to encompass a total of 1,176 images. Scaling factors were randomly generated between 0.95 and 1.05, while rotation angles were randomly generated from −5° to +5°. The YOLO v5 model was used to extract brainstem regions from the augmented dataset, with an extracted region size of 40 × 40 × 4.

As the pathological features in the brainstem images obtained were not readily discernible, we further enhanced the images through greyscale inversion and greyscale equalization, as shown in the Brainstem Image Obtaining section of Figure 3. Gray scale inversion is achieved by determining the maximum greyscale value in each image and subtracting this value from the greyscale value of each pixel. This inversion process prominently broadened the greyscale distribution in the image, thereby enhancing the image contrast to a certain extent. Subsequently, a greyscale equalization strategy was employed to improve the image contrast further.

#### 2.4.2 Construction and training of the CNN

Building upon LeNet architecture, we introduced enhancements to the network, as outlined in the Parkinson’s Disease Classification section (Figure 3). In this architecture, a greyscale image with a size of 40 × 40 served as input, sequentially traversing two sets of convolutional and max-pooling layers. Subsequently, it is flattened into a 1 × 200 feature vector in the final fully-connected layer, followed by a softmax operation to obtain the predicted class of the input image. The structural details are described in Supplementary material S4.

The training-validation set underwent training using a five-fold cross-validation approach, with evaluation conducted on the test set. Details of model parameter settings were provided in Supplementary material S5. An evaluation function for model performance was established in each epoch to assess the accuracy of the model on the validation subset, and the training model with the highest metrics during the training process was recorded and saved.

### 2.5 Hybrid features for diagnosis of PD

Radiomics features primarily encompass greyscale statistics and texture features, while deep learning features extracted via CNNs emphasize specific higher-order semantic information. Recognizing this, we hypothesized that the two approaches may be complementary for the classification task. Hybrid features contained both radiomics lower-order features and deep learning higher-order features. The flow chart is shown in Figure 2 Hybrid model. To reduce the dimensionality of features extracted at the fully connected layer of the CNN model, feature filtering was necessary. We quantified feature importance using the Mean Decrease in Impurity (MDI) evaluation method (Supplementary material S6) based on the random forest classifier. This involved running the random forest algorithm 100 times and ranking feature importance after averaging. The top 10 features were selected and combined with the radiomics features extracted in section 3.2 to form a new feature vector. Finally, this new feature vector was utilized to model the classifiers using the same methodologies as outlined in Section 3.2, employing six machine learning methods.

### 2.6 Statistical analysis

The statistical analysis was performed using Stata/SE software,^4^ and Python^5^ environment. In Stata/SE software, appropriate statistical tests were applied to demographical characteristics. Numerical variables were analyzed using the Mann–Whitney U-test, while categorical variables were assessed using the Pearson’s *χ*^2^-test. A two-sided *p*-value less than 0.05 was considered to indicate statistical significance. Radiomics feature extraction, feature selection, model training and testing were performed in Python environment. The training of the CNN model was implemented on a Windows 10 platform equipped with a GeForce RTX 3080 GPU based on Tensorflow.

## 3 Results

### 3.1 Clinical characteristic of the patients

In Center 1, a total of 65 cases of HC and 73 cases of PD were included in this study. There were no significant differences in age and sex between the patient and healthy control groups. 30 male and 35 female individuals were in the HC group, with the mean age of 57.82 ± 6.26 years ranging from 43 to 69. And 40 male and 33 female patients were in the PD group, with the mean age of 59.11 ± 8.78 years ranging from 37 to 78. The mean Hoehn-Yahr stage in the PD group was 1.72, with a variance of 0.45. As for Center 2, the images of 24 participants were collected as an external validation set, including 12 HCs and 12 PDs. The detailed information of demographic and clinical information were summarized in Table 1.

**Table 1:** Demographic and clinical information of HC and PD.

Variables	HC (*n* = 65)	PD (*n* = 73)	*p*-value	HC_E (*n* = 12)	PD_E (*n* = 12)	*p*-value
Gender(male/female)	30:35	40:33	0.39	6:6	7:5	>0.99
Age	57.82 ± 6.26	59.11 ± 8.78	0.33	53.58 ± 4.64	56.92 ± 13.19	0.42
Hoehn-Yahr stage	–	1.72 ± 0.45	–	–	1.92 ± 0.29	–
DD(months)	–	24.11 ± 20.22	–	–	35.88 ± 16.52	–
UPDRS-III	–	21.49 ± 11.73	–	–	37.13 ± 8.95	–
tremor	–	3.46 ± 3.60	–	–	5.00 ± 6.80	–
rigid	–	3.09 ± 2.62	–	–	8.88 ± 3.40	–
bradykinesia	–	11.38 ± 6.96	–	–	19.38 ± 4.81	–

DD, disease duration; UPDRSIII, Unified Parkinson’s Disease Rating Scale part III. Results are expressed as mean ± standard deviation; HC_E, healthy controls in external validation set; PD_E, Parkinson’s disease patients in external validation set.

### 3.2 The performance of radiomics classification

**Figure 4:**
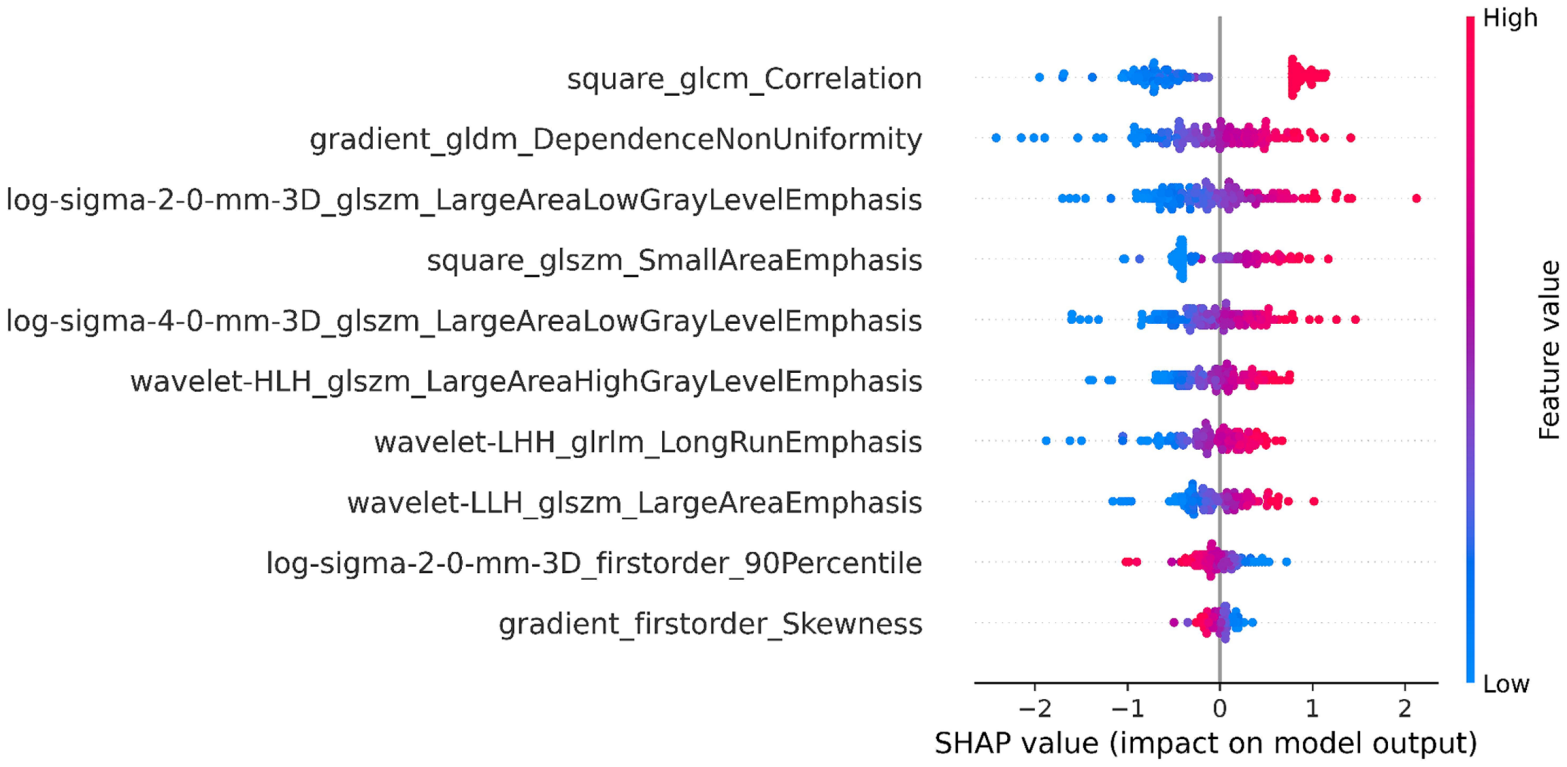
SHAP value-based interpretation of the model, showing the importance of contributing features. The blue and red points in each row represent participants with low to high values for each specific variable.

**Figure 5:**
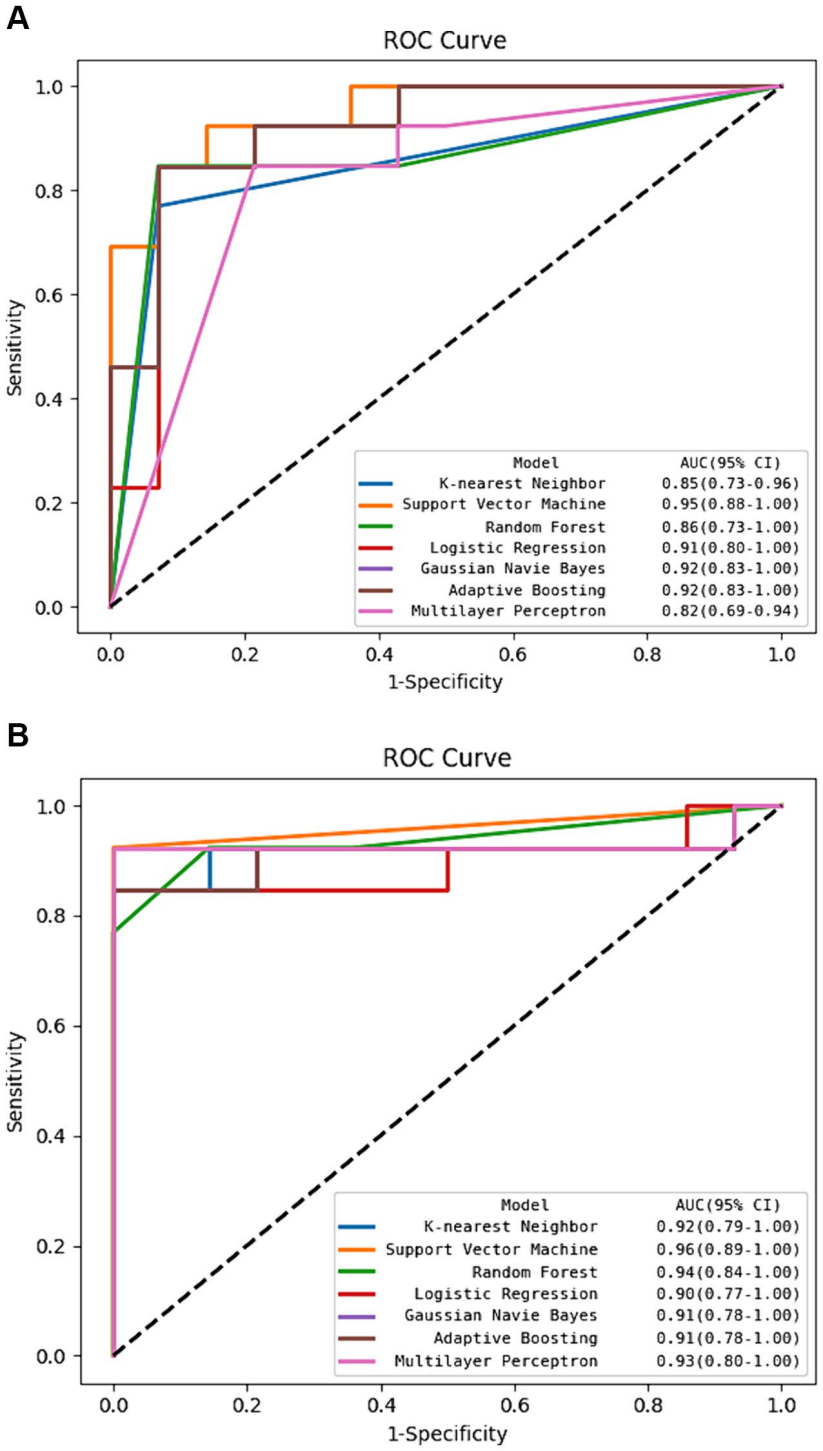
The ROC curve of machine learning algorithms in the test set. **(A)** Using radiomics features alone. **(B)** Using hybrid features.

The DSC of the ROIs segmented by two different radiologists was excellent (DSC = 0.925 ± 0.019). In total, 1781 radiomics features were taken into consideration. After ICC and LASSO filtering and selection, 10 crucial radiomics features remained (Supplementary Table S1). Six machine learning (ML) models were trained and tested using these features. The results of the test set are shown in Table 2, and the overall classification performance is relatively average. The best-performing model is SVM: ACC = 0.889, SEN = 0.923, SPE = 0.857, PPV = 0.857, NPV = 0.923, F1-score = 0.889, AUC = 0.95 (0.88–1.00) The parameter settings for the six ML models and results of the five-fold cross training-validation were shown in Supplementary materials S5, S7. As shown in Figure 4, the conclusion of the shap analysis performed on the SVM model is consistent with the feature screening process, which means that the 10 features selected have a large contribution to Parkinson’s classification. In addition, we plotted and calculated the ROC curves and AUC for each machine learning method (Figure 5).

**Table 2:** The performance of each method on the test set.

Feature	Model	ACC	SEN	SPE	PPV	NPV	F1-score	NRI	IDI
Radiomics features	KNN	0.852	0.929	0.769	0.813	0.909	0.833	–	
	SVM	**0.889**	**0.923**	**0.857**	**0.857**	**0.923**	**0.889**		
	RF	0.852	0.769	0.929	0.909	0.813	0.833		
	LR	0.815	0.769	0.857	0.833	0.800	0.800		
	AdaBoost	0.852	0.769	0.929	0.909	0.813	0.833		
	MLP	0.778	0.846	0.7714	0.7733	0.8333	0.788		
Deep features	CNN	**0.926**	**0.923**	**0.929**	**0.923**	**0.929**	**0.923**	–	
Hybrid features	KNN	0.926	0.923	0.929	0.923	0.929	0.923	0.192 (*p* = 0.583)	0.541 (*p* = 0.020)
	SVM	**0.963**	**0.923**	**1.000**	**1.000**	**0.9333**	**0.960**	**0.218 (*p* = 0.245)**	**0.153 (*p* = 0.112)**
	RF	0.889	0.923	0.857	0.857	0.923	0.889	0.126 (*p* = 0.685)	0.175 (*p* = 0.048)
	LR	0.852	0.846	0.857	0.846	0.857	0.846	0.092 (*p* = 0.436)	0.184 (*p* = 0.047)
	AdaBoost	0.926	0.923	0.929	0.923	0.929	0.923	0.284 (*p* = 0.069)	0.127 (*p* = 0.162)
	MLP	0.926	0.846	0.857	0.846	0.857	0.846	0.084 (*p* = 0.699)	0.178 (*p* = 0.203)

Bold values highlight the models with the highest ACC.

### 3.3 The performance of CNN classification

We improved the LeNet model for classification. The model was trained and tested using the image data of the brainstem extracted by the YOLO v5 model. The performance of the YOLO v5 model is shown in Supplementary material S8; Supplementary Figure S1. When the test output was stable, the following results were obtained: Loss = 0.297 and Accuracy = 0.995. As shown in Table 2, the classification performance of the CNN compared well with the machine learning algorithm trained on the radiomics features, and accuracy has reached above 0.9.

### 3.4 The performance of feature fusion and classification

**Figure 6:**
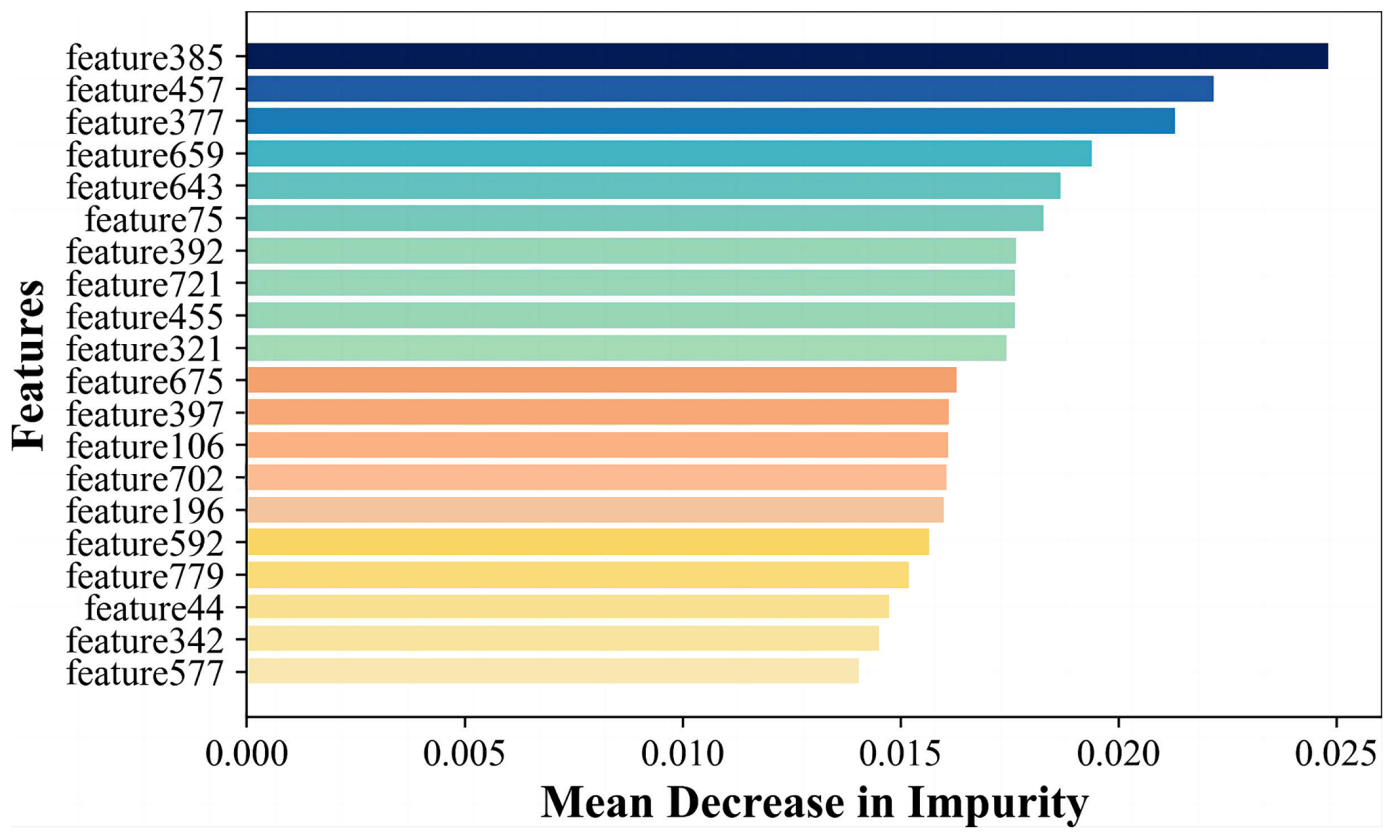
The average importance of the features in the random forest.

In order to further enhance classification performance, the features of both radiomics and CNN approaches were then filtered and fused. We input 4 images of each case into the CNN model and extract features in the final fully-connected layer to obtain vector features of size 4 × 200 corresponding to each case. To fuse with radiomics features, we calculated and ranked the importance of the features using the random forest algorithm, as shown in Figure 6, and selected the top 20 features for fusion with the radiomics features according to the ranking. Finally, we obtained a total of 138 cases with a feature vector of size 1 × 30 for each case.

The classification performance based on hybrid features is show in Table 2 and Figure 5, and the overall metric level is improved compared with the radiomics features alone. Basically, every model achieved an accuracy of around 90%, with the SVM model improving the most: ACC = 0.963, SEN = 0.923, SPE = 1.000, PPV = 1.000, NPV = 0.933, F1-score = 0.960, AUC = 0.96 (95% CI: 0.89–1.00). Moreover, NRI and IDI showed that the classification ability of the model constructed based on hybrid features has been improved. Among these, NRI and IDI indicated that the SVM model with the improvement by 0.245 and 0.112, respectively.

### 3.5 The performance of the external validation set

To demonstrate the generalization ability of the proposed model, we applied the radiomics feature-based support vector machine (SVM) model, the image-based deep learning model, and the hybrid feature-based SVM model on the external validation set. The detailed results are shown in Table 3. The performance of the hybrid feature-based SVM is satisfactory, with ACC = 0.958, SEN = 1.000, SPE = 0.933, PPV = 0.900, NPV = 1.000, and F1-score = 0.947.

**Table 3:** The performance of optimal models on the external validation set.

Feature	Model	ACC	SEN	SPE	PPV	NPV	F1-score
Radiomics features	SVM	0.875	0.889	0.867	0.800	0.929	0.842
Deep features	CNN	0.917	0.929	0.900	0.929	0.900	0.900
Hybrid features	SVM	0.958	1.000	0.933	0.900	1.000	0.947

### 3.6 Feature statistics and visualization

**Figure 7:**
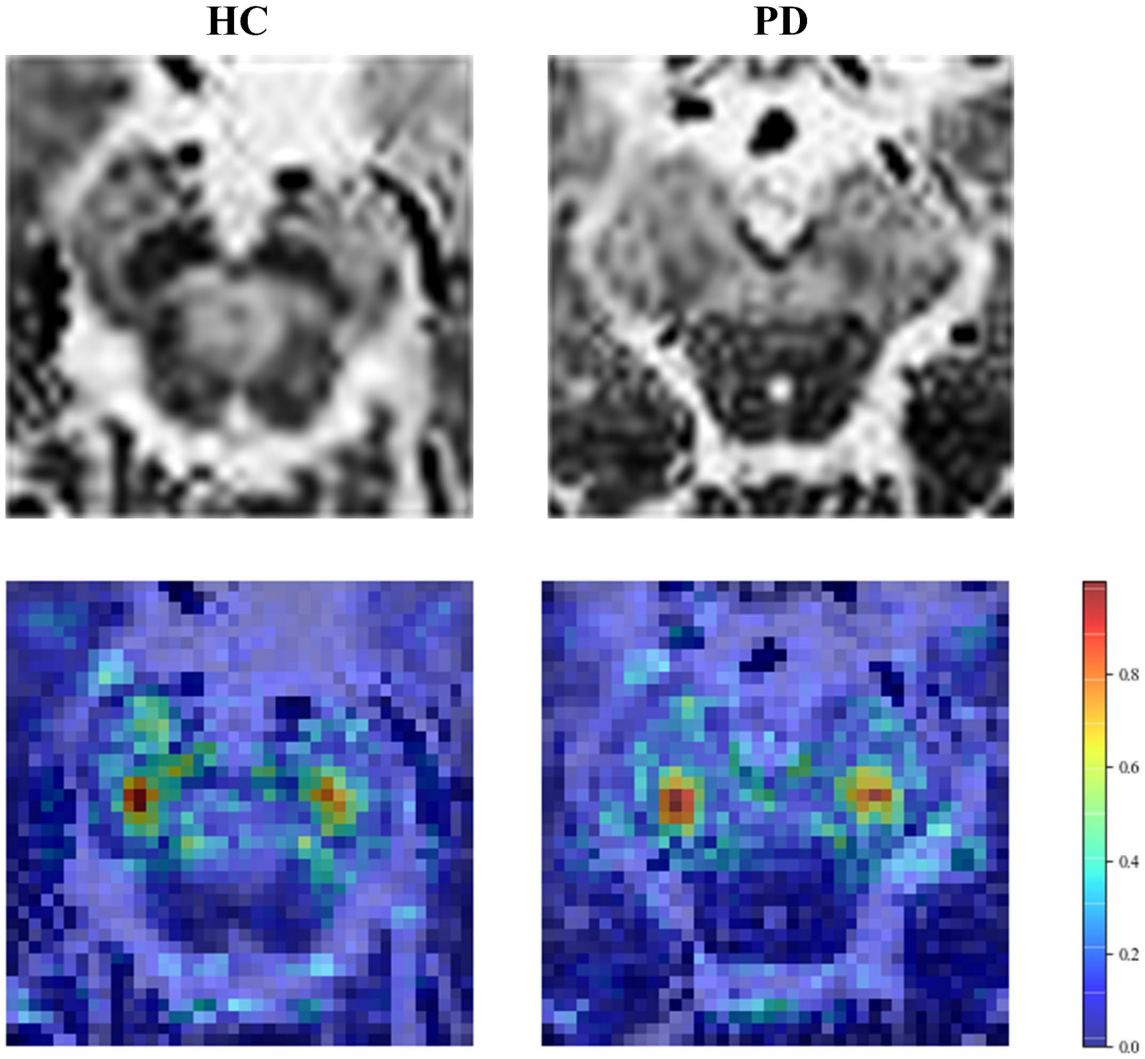
Saliency map of the HC and PD case. The first row is the original image, and the second row is the saliency map.

The accuracy of our model was further improved by fusing deep learning features. To explain the role of deep learning features, we visualized regions that influence classification decisions via a saliency map [23], which principles are explained in Supplementary material S9. As shown in Figure 7, the focus of the saliency map was on the bilateral “swallow tail” sign, a characteristic of PD, which was consistent with the regions of pathological change, greatly enhancing the interpretability of our CNN model.

## 4 Discussion

We proposed a novel hybrid model utilizing combined features from both deep learning and radiomics to identify early-stage PD. Utilizing the setMag images reconstructed from QSM images, we can synchronously extract information such as neuromelanin (NM) and iron deposition without increasing scan time. This approach can provide valuable insights for future research on the pathological changes associated with PD. In this study, the YOLO v5 model was used to segment the brainstem automatically. Then an improved lightweight LeNet model was used to diagnose the brainstem and provide deep learning features. The combination of radiomics features and deep learning features improved the accuracy of machine learning models. This integrative image analysis strategy is more sensitive to the initial changes in neuromelanin density and volume, providing distinct radiomics information and deep features hidden within setMag images. The proposed approach would be helpful in improving the diagnostic accuracy for EPD and increasing the confidence of clinicians in the diagnosis, especially when supplemented with clinical information.

In our opinion, this study had three improvements over similar studies to date. Firstly, we focused on the features of the brainstem, where the pathological changes in PD mainly occurred. Although using the whole-brain textural features has been the main focus of previous studies to classify PD patients and healthy people (Wu et al., 2019; Shi et al., 2022), the development of models based on the brainstem is showing significant diagnostic potential. For instance Haq et al. (2020) produced positive results, and Huddleston et al. (2018) achieved an accuracy of 86% in the machine learning model using features from the SNpc of the brainstem. In our study, segmenting brainstem areas and training the model with hybrid features led to a discriminative diagnosis of early-stage PD with accuracy of 96.3% in the test set and 95.8% in the external validation set.

Secondly, we used a new imaging MRI modality, a simple CNN model, and a visualization technique to complete the classified diagnosis. To the best of our knowledge, modalities such as NM-MRI (Le Berre et al., 2019), QSM (Xiao et al., 2019), and SPECT (Pahuja and Prasad, 2022) have provided major information for deep learning methods in PD. Large and complicated deep learning models were typically used to extract higher-order semantic features from images. To ensure classification accuracy, manual annotation, data augmentation, and transfer learning were all frequently used. We obtained neuromelanin content based on setMag images, which had been reported for its extension value of QSM in our previous study (Liu et al., 2020). We also confirmed the efficacy of this imaging technique in detecting early-stage Parkinson’s disease. In addition, we employed an improved LeNet model, which not only requires less computation and training data but also effectively avoids gradient disappearance problems and local optimal solutions. In the visualization result of the saliency map, the highlighted parts are positively correlated with the classification decision of the model. We found the highlighted regions were primarily within the bilateral SNpc, which was in line with the findings of other researchers (Blazejewska et al., 2013; Gao et al., 2016; Schwarz et al., 2017). As a result, the features extracted by the CNN appear to reflect some of the pathological changes caused by PD, improving the interpretability and diagnostic value of the model.

Finally, in comparison to the research employing only deep learning methods, the combination of radiomics features and deep learning features improved the diagnosis of PD. In a recent study Sun et al. (2022) achieved 85.58% accuracy using the ResNet34 model for image coding and SVM to classify early-stage PD. In another attempt Xiao et al. (2019) obtained a 90% classification accuracy using a network structure with only three convolutional layers. Since deep learning approaches can extract higher-order semantic information, while radiomics approaches can provide information on gray-scale statistics and texture features, combining both can increase diagnostic accuracy and outperform similar recent research (Sun et al., 2022) in diagnostic value.

There were several limitations in this study. Firstly, the proposed CNN model used a single slice of a 2D brainstem region, which lost the spatial information of the original image. It would be better to develop models that can input 3D images to exploit spatial contextual information. Second, our training data was only from one clinical center. Unicentric studies could be biased with the population of the study. Besides, the sample size is still relatively small for machine learning. Although we evaluated the model in an external validation set, the model still requires more available external validation to further verify the generalizability and robustness of the diagnostic model, as well as its interpretability.

In conclusion, we proposed a hybrid machine learning model with deep learning features and radiomics features, improved the diagnostic ability for detecting early PD, and demonstrated the interpretability by different locations of the brainstem showing different levels of significance. Our results suggest that the use of the hybrid model can identify Parkinson’s disease with high accuracy. The hybrid approach may provide a reliable suggestion for the early detection of Parkinson’s disease to help clinicians make accurate decisions.

## Data availability statement

The original contributions presented in this study are included in this article/Supplementary materials, further inquiries can be directed to the corresponding author.

## Ethics statement

This study was approved by the Ethics Committee of Huashan Hospital, Fudan University (approval No. KY 2016–214). All patients or their guardians gave their informed consent to the utilization of their anonymized MRI images and clinical data for research purposes.

## Author contributions

HC: Writing – original draft, Writing – review & editing, Funding acquisition. XuL: Data curation, Writing – original draft. XiL: Writing – review & editing. JF: Data curation, Writing – review & editing. KZ: Writing – review & editing. NW: Writing – review & editing. YL: Writing – review & editing. DG: Writing – review & editing.

## Footnotes

^1^ www.itksnap.org

^2^ https://afni.nimh.nih.gov/

^3^v. 3.0.1; http://www.radiomics.io/pyradiomics.html

^4^Version 16.0, http://www.stata.com

^5^Version 3.8.0, https://www.python.org
