# Supplementary material for: An automated hybrid approach via deep learning and radiomics focused on the midbrain and substantia nigra to detect early-stage Parkinson’s disease
Source: Front Aging Neurosci. 2024 May 20;16:1397896. doi: 10.3389/fnagi.2024.1397896 (PMC11144908; doi:10.3389/fnagi.2024.1397896)
Supplement: Supplementary file 1 [file Data_Sheet_1.PDF]

Supplemental Materials

S1. Inclusion criteria

Patients who meet the following inclusion criteria were collected into this study:

1)Parkinson's disease patients: a) a diagnosis of PD according to the Movement Disorder Society Clinical Diagnostic Criteria[1]; b) onset age ⩾40; and c) early-stage PD with a Hoehn and Yahr stage (H-Y) ⩽2.

2)Healthy control group: a) Age > 40；b) No family history of neurological or psychiatric disorders；c) No PD prodromal-related motor or non-motor system symptoms (e.g., sleepwalking, hyposmia, constipation, etc.) d) No contraindications to MRI examinations such as claustrophobia.

The severity of motor dysfunction was assessed based on the Movement Disorder Society Unified Parkinson's Disease Rating Scale (MDS-UPDRS) Part III[2], and we calculated the sub-scores of rigidity (items 3.3), bradykinesia (items 3.4~8) and tremor (items 3.15~17). Disease duration was calculated from the time of clinical symptoms onset to the time of brain MRI acquisition (by month).

S2. MRI procedures

All MR examinations were performed on a 3.0-T MR scanner (Signa HDxt; GE 750 Medical Systems, Milwaukee, Wis, USA) equipped with an eight-channel head matrix coil at the Department of Radiology of Huashan Hospital of Fudan University, and 3.0T MR750W scanner (GE Healthcare, Milwaukee, WI) in Weifang Traditional Chinese Hospital. Foam padding was applied to prevent head movement for each participant, and earplugs were provided to reduce scanner noise.

QSM images were performed with a 3D multi-echo gradient recalled echo sequence (TR/TE/ΔTE: 41.6/3.2/2.4 ms, TE(16 group TEs): 3.2, 5.5, 7.9, 10.2, 12.6, 15.0, 17.3, 19.7, 22.0, 24.4, 26.7, 29.1, 31.4, 33.8, 36.1, 38.5 ms, bandwidth: 62.5 kHz; flip angle: 12°, field of view: 256 × 256 mm, acquisition matrix size: 512 × 512/ 256 × 256, slice thickness: 1 mm, spacing: 0.5×0.5×1mm/1×1×1mm, number of slice: 140 (no sap, interleaved), scan time: 9:00min). In addition, in order to exclude other pathological diseases in the mesencephalon, conventional MRI scans including T2-weighted fluid-attenuated inversion recovery (FLAIR) and diffusion-weighted images (DWI) were also acquired prior to the QSM sequence. All sequences were acquired in the axial plane parallel to the anterior commissure–posterior commissure line (or AC-

PC line).

S3. Evaluation Metrics

The model was evaluated using multiple metrics during training and validation: accuracy(ACC), sensitive(SEN), specificity(SPE), positive predictive value(PPV), negative predictive value(NPV), F1-Score, net reclassification improvement(NRI) and integrated discrimination improvement(IDI). The formula is shown in equations (1)-(8):

(1).ACC = (TP+TN)/(TP+TN+FP+FN)

(2).SEN = TP / (TP + FN)

(3).SPE = TN / (TN + FP)

(4).PPV/Precision = TP/(TP+FP)

(5)NPV = TN/(TN+FN)

$$(6).F1-Score \ = \frac{2*(TP \ / \ (TP+FP))(TP \ / \ (TP+FN))}{(TP \ / \ (TP+FP)+TP \ / \ (TP+FN))}$$

$$(7).NRI \ = (SEN_{new}+SPE_{new})-(SEN_{old}+SPE_{old})$$

$$(8).IDI \ = (P_{new,PD}+P_{old,PD})-(P_{new,HC}-P_{old,HC})$$

Note: TP=true positive, TN=true negative, FP=false positive, FN=false negative, SEN_{new} = SEN of the new model, $SEN_{old} = SEN$ of the old model, SPE_{new} = SPE of the new model, SPE_{old} = SPE of the old model, $P_{new,PD}$ = the average value of PD occurrence probability predicted by the new model in the PD group, $P_{new,HC}$ = the average value of PD occurrence probability predicted by the new model in the HC group, $P_{old,PD}$ = the average value of PD occurrence probability predicted by the old model in the PD group, $P_{old,HC}$ = the average value of PD occurrence probability predicted by the old model in the HC group.

S4. Convolutional neural network structure Model parameter setting

Our convolutional neural network was inspired by the classical LeNet-5. The improved model network architecture is 6 layers taking into account the softmax output layer. The specific structure of the model is as follows: a first convolution layer(shape: 40×40×20), a first max pooling layer(shape: 20×20×20), a second convolution layer(shape: 20×20×50), a second max pooling layer(shape: 10×10×50), a fully connected layer(shape: 200) and the softmax output layer(shape: 2). Besides, a flatten operation was used between the second max pooling layer and the output layer.

S5. Model parameter setting

In the CNN classification experiment, the CNN model was optimized using the Adam algorithm using categorical cross-entropy as the loss function. The initial epoch was set to 100, the batch size to 8, and the initial learning rate to 0.0001.

In the radiomics classification and feature fusion and classification experiment, the parameters for training the six machine learning models were set as following:

```
KNN = KNeighborsClassifier(n_neighbors=2, weights='distance', p=1)
SVM = SVC(kernel='rbf', gamma='auto', C=1, decision_function_shape='ovo',probability=True, tol=1e-1,random_state=1)
RF = RandomForestClassifier(n_estimators=3, criterion='entropy', max_depth=5)
LR = LogisticRegression(C=0.1)
GNB = GaussianNB(priors=None, var_smoothing=1e-30)
AB = AdaBoostClassifier(
    DecisionTreeClassifier(max_depth=3),
    n_estimators=100,
    learning_rate=1,
    algorithm="SAMME")
MLP = MLPClassifier(solver='lbfgs', alpha=1e-8, max_iter=2000, tol=1e-13)
```

S6. Mean Decrease in Impurity (MDI) evaluation method

MDI is a metric that measures the impact of each feature on the classification results in a random forest, obtained by calculating the degree to which the accuracy of the model classification is reduced when a feature is removed. We ran the random forest algorithm 100 times and ranked their importance after averaging. Supplemental Figure II shows the top 20 features and their MDI values. The formula is shown in equation (9):

$$(9).\,\mathrm{MDI(X)} = \frac{1}{N_T}\sum_{T}\sum_{t\in T:v(s_t)} p(t)\Delta i(s_t, t)$$

where t is the node in the tree T, $v(s_t)$ denotes the variable tested at node t, $p(t)$ is the fraction of samples reaching node t, $\Delta i(s_t, t)$ is the metric using the Gini index as the impurity function and N_T is the quantity of trees in forests. By summing over all interior nodes in T and averaging over all trees, weighted impurity decreases quantifying the importance of a feature X on the classification results.

S7. The results on the training set

Feature	Model	ACC	SEN	SPE	PPV	NPV	F1-score
Radiomics features	KNN	0.896±0.110	0.955±0.307	0.846±0.305	0.840±0.279	0.957±0.286	0.894±0.314
	**SVM**	**0.913±0.162**	**0.929±0.169**	**0.889±0.128**	**0.929±0.139**	**0.889±0.164**	**0.929±0.159**
	RF	0.870±0.274	1.000±0.283	0.667±0.276	0.824±0.284	1.000±0.395	0.903±0.305
	LR	0.826±0.307	0.929±0.311	0.667±0.313	0.813±0.314	0.857±0.327	0.867±0.374
	AdaBoost	0.870±0.247	0.786±0.274	1.000±0.264	1.000±0.259	0.750±0.228	0.880±0.215
	MLP	0.875±0.139	0.909±0.162	0.846±0.169	0.833±0.128	0.917±0.159	0.870±0.164
Deep features	**CNN**	**0.957±0.101**	**0.929±0.083**	**1.000±0.128**	**1.000±0.119**	**0.900±0.096**	**0.963±0.093**
Hybrid features	KNN	0.947±0.129	0.966±0.127	0.923±0.124	0.959±0.114	0.938±0.121	0.953±0.098
	**SVM**	**0.986±0.072**	**0.953±0.085**	**1.000±0.082**	**1.000±0.091**	**0.970±0.086**	**0.992±0.114**
	RF	0.910±0.176	0.957±0.156	0.909±0.183	0.914±0.201	0.926±0.231	0.938±0.183
	LR	0.873±0.103	0.936±0.126	0.962±0.133	0.904±0.174	0.907±0.158	0.912±0.198
	AdaBoost	0.952±0.105	0.938±0.089	0.942±0.097	0.959±0.063	0.962±0.075	0.978±0.110
	MLP	0.961±0.188	0.973±0.194	0.939±0.176	0.962±0.135	0.958±0.128	0.984±0.198

S8. Brain stem image extraction

For YOLO v5, the ratio of training and testing data was 8:2. We annotated the brain MRI images used for training. Supplemental Figure I shows the changes in model performance during training for 100 epochs. When the loss tended to stabilize, the best performance of the model on the validation set reached box_loss = 0.016, obj_loss = 0.004, mAP_0.5 = 0.995, mAP_0. 5:0.95 = 0.870. Using the trained model to process the brain MRI dataset, a total of 121 cases containing 484 brainstem images were obtained. It can be seen that the YOLO v5 model has a better detection effect on the brainstem area, and can accurately extract the brainstem data to provide a basis for subsequent experiments. (Supplemental Figure I)

S9. CNN visualization—saliency map

To clarify and explain the reliability of the CNN model features, we used the

saliency map to visualize the extracted fully connected layer features. This method provides an explanation for image classification by backpropagation to compute and determine the support decision regions in a given image for the predicted category. Assuming an image P_o belonging to category c, by calculating the category score function $S_c(P_o)$ we can rank the pixels in $P_o$according to their influence on $S_c(P_o)$. By using the first-order Taylor expansion we can approximate the category score function in the CNN with a linear equation:

$$(10). S_c(P_o) \approx \omega_c^T P + b_c$$

where b_c is the bias and ω_c is the weight of the model which is the derivative of S_c at the image P_o.

$$(11). \omega_c = \frac{\partial S_c}{\partial P} \mid_{P_0}$$

Finally, the weights ω_c are further smoothed using the Gaussian kernel-based method proposed by Smilkov et al[3]. This operation removed the effect of noise in the weights, thus further highlighting the concerns of the convolutional network model embodied in the saliency map.

Supplemental Table

Supplemental Table1. Ten selected radiomic features

Filters	Radiomic features	
Square	GLCM	Correlation
Square	GLSZM	SmallAreaEmphasis
Gradient	GLDM	DependenceNonUniformity
Gradient	FIRSTORDER	Skewness
log-sigma-2-0-mm-3D	GLSZM	LargeAreaLowGrayLevelEmphasis
log-sigma-2-0-mm-3D	FIRSTORDER	90Percentile
wavelet-HLH	GLSZM	LargeAreaHighGrayLevelEmphasis
wavelet-LHH	GLRLM	LongRunEmphasis
wavelet-LLH	GLSZM	LargeAreaEmphasis
log-sigma-4-0-mm-3D	GLSZM	LargeAreaLowGrayLevelEmphasis

Note: These features included 2 first-order features, 1 grey-level co-occurrence matrix (GLCM) feature, 1 gray-level run-length matrix (GLRLM) feature, 5 gray-level size zone matrix (GLSZM) features, and 1 gray level dependence matrix (GLDM) feature.

Supplemental Figures

Supplemental Figure I. Performance of the YOLO v5 model.

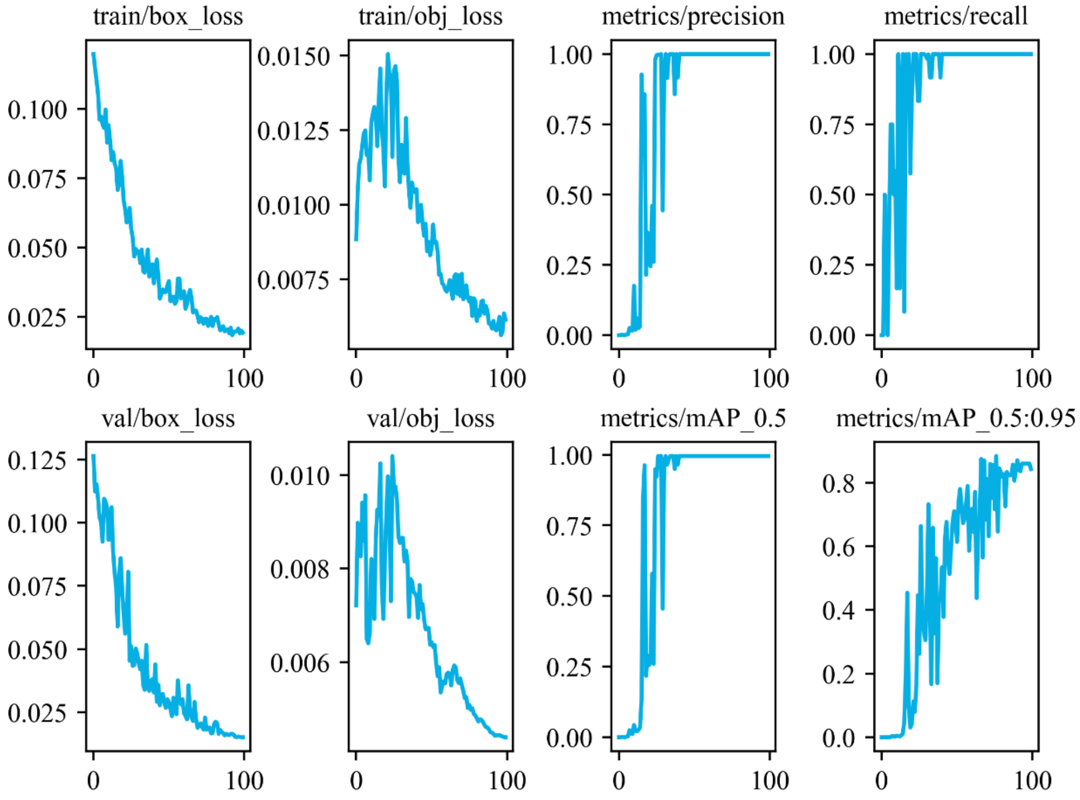

Reference

[1] Postuma RB, Berg D, Stern M et al (2015) MDS clinical diagnostic criteria for Parkinson's disease. 30:1591-1601

[2] Goetz, C.G., et al., Movement Disorder Society-sponsored revision of the Unified Parkinson's Disease Rating Scale (MDS-UPDRS): scale presentation and clinimetric testing results. Mov Disord, 2008. 23(15): p. 2129-70.

[3] Smilkov D, Thorat N, Kim B, Viégas FB, Wattenberg M (2017) SmoothGrad: removing noise by adding noise. ArXiv abs/1706.0382
